# Towards IVDR‐compliance by implementing quality control steps in a quantitative extracellular vesicle‐miRNA liquid biopsy assay for response monitoring in patients with classic Hodgkin lymphoma

**DOI:** 10.1002/jex2.164

**Published:** 2024-06-28

**Authors:** Esther E. E. Drees, Nils J. Groenewegen, Sandra A. W. M. Verkuijlen, Monique A. J. van Eijndhoven, Jip Ramaker, Pepijn Veenstra, Mirjam Hussain, Catharina G. M. Groothuis‐Oudshoorn, Daphne de Jong, Josée M. Zijlstra, Johan de Rooij, D. Michiel Pegtel

**Affiliations:** ^1^ Department of Pathology Amsterdam UMC, Location Vrije Universiteit Amsterdam Amsterdam The Netherlands; ^2^ Cancer Center Amsterdam Program Imaging and Biomarkers Amsterdam The Netherlands; ^3^ ExBiome B.V. Amsterdam The Netherlands; ^4^ Department of Health Technology and Services Research Technical Medical Centre, University of Twente Enschede The Netherlands; ^5^ Department of Hematology Amsterdam UMC, Location Vrije Universiteit Amsterdam Amsterdam The Netherlands

**Keywords:** extracellular vesicles (EVs), Hodgkin lymphoma, in vitro diagnostics (IVD), liquid biopsy, microRNA, quality control (QC), response monitoring

## Abstract

Previously, we showed that quantification of lymphoma‐associated miRNAs miR‐155‐5p, ‐127‐3p and let‐7a‐5p levels in plasma extracellular vesicles (EVs) report treatment response in patients with classic Hodgkin lymphoma (cHL). Prior to clinical implementation, quality control (QC) steps and validation are required to meet international regulatory standards. Most published EV‐based diagnostic assays have yet to meet these requirements. In order to advance the assay towards regulatory compliance (e.g., IVDR 2017/746), we incorporated three QC steps in our experimental EV‐miRNA quantitative real‐time reverse‐transcription PCR (q‐RT‐PCR) assay in an ISO‐13485 certified quality‐management system (QMS). Liposomes encapsulated with a synthetic (nematode‐derived) miRNA spike‐in controlled for EV isolation by automated size‐exclusion chromatography (SEC). Additional miRNA spike‐ins controlled for RNA isolation and cDNA conversion efficiency. After deciding on quality criteria, in total 107 out of 120 samples from 46 patients passed QC. Generalized linear mixed‐effect modelling with bootstrapping determined the diagnostic performance of the quality‐controlled data at an area under the curve (AUC) of 0.84 (confidence interval [CI]: 0.76–0.92) compared to an AUC of 0.87 (CI: 0.80–0.94) of the experimental assay. After the inclusion of QC steps, the accuracy of the assay was determined to be 78.5% in predicting active disease status in cHL patients during treatment. We demonstrate that a quality‐controlled plasma EV‐miRNA assay is technically robust, taking EV‐miRNA as liquid biopsy assay an important step closer to clinical evaluation.

## 1 INTRODUCTION

Extracellular vesicles (EV) associated RNAs have great potential as biomarkers for disease detection and for monitoring response to therapy in solid cancer (Chen et al., 2018; Cordonnier et al., 2020; Drees et al., 2021; Rajakumar et al., 2022) and malignant lymphoma (Drees & Pegtel, 2020; Drees et al., 2021; Eijndhoven et al., 2016; Manier et al., 2017). Thus far, only one EV‐based assay (prostate cancer detection in urinary EVs) has coverage by health maintenance organizations (HMOs) (McKiernan et al., 2016) and has been assigned Food and Drug Administration (FDA) breakthrough designation in 2019 (Clair, 2019; Kretschmer et al., 2022). Compared to the large number of publications with EVs as promising liquid biopsy source, the pace of implementation into clinical practice is disappointing (Drees & Pegtel, 2020). Even though the techniques and biomarkers may be available in research settings, inadequate pre‐analytical parameters and insufficient technical validation preclude such advancement. Initiatives, such as CANCER‐ID (Rossi et al., 2020), Minimal Information for Studies of Extracellular Vesicles (MISEV) criteria (Théry et al., 2018; Witwer et al., 2021), Minimum Information for Publication of Quantitative Real‐Time PCR Experiments (MIQE) guidelines (Bustin et al., 2009) and Blood Profiling Atlas in Cancer (BLOODPAC) (Leiman et al., 2022) may accelerate clinical application by standardizing pre‐analytical, technical parameters and uniform reporting. Nevertheless, rigorous technical validation and implementation of biomarker‐assays guided by quality management system (QMS) with standard operating procedures (SOPs) are required. Currently, few cell‐free based miRNA assays reached this stage, with a few notable exceptions (Khamina et al., 2022; McKiernan et al., 2016). Generally, the “signal‐to‐noise” (STN) ratio needs to be in favour of the biological signal and technical noise should be recognized, quantified, minimized and/or controlled. The STN ratio for biomarkers depends on numerous biological and technical variables, as we reviewed previously (Drees & Pegtel, 2020).

To gain confidence in EV‐based liquid biopsy assays, rigorous technical validation and comprehensive quality control (QC) is required to meet regulatory standards, in particular the EU Regulation 2017/746 on In Vitro Diagnostic medical devices (IVDR). We previously developed a plasma EV‐miRNA detection assay making use of size‐exclusion chromatography (SEC) to isolate bulk EVs from blood plasma and subsequent quantitative real‐time reverse‐transcription PCR (qRT‐PCR) for quantification of a panel of EV‐bound miRNAs. With this assay, we could determine disease activity as measured by 18F‐fluorodeoxyglucos‐positron emission tomography/computed tomographic (FDG‐PET/CT) imaging in patients with classic Hodgkin lymphoma (cHL) (Drees et al., 2021). Here, we used an ISO‐13485 certified QMS to implement quality controls at all steps of the assay. The QC‐steps enabled us to quantify the technical noise generated during the EV‐isolation, RNA extraction and qRT‐PCR steps while retaining confidence in the biologically motivated signal associated with disease activity. Implementation of QC‐steps increased confidence in the diagnostic performance of the assay, taking quantitative measurements of EV‐miRNAs a step closer to clinical application.

## 2 MATERIAL AND METHODS

### 2.1 Sample collection and selection

Blood samples were prospectively collected in the Amsterdam UMC location of the BioLymph‐study and a single institution Biobank cohort. BioLymph‐study is a multicentre study in region Amsterdam (the Netherlands) that longitudinally collects blood samples from lymphoma patients. From both these cohorts, 120 samples (pre‐, during and post‐treatment and a maximum of three samples per patient) from 51 cHL patients were selected for analyses. Selection criteria were: histologically confirmed cHL according to the revised 4th edition of the World Health Organization (WHO) classification for Haematolymphoid tumours (2016) (Sud et al., 2017), older than 18 years. Patients with other malignancies that require treatment simultaneously with the treatment of the lymphoid malignancy were not selected. All patients included were from the same single centre (i.e., Amsterdam UMC, location VUmc) and processed under the same laboratory conditions. This cohort was used to test the QC‐approved cHL EV‐miRNA assay. Other samples from the same cohorts were used for the technical optimization experiments prior to performing a design freeze on the EV‐miRNA cHL assay in QC setting. Blood from one healthy donor was collected via biobanking for one of the technical optimization experiments.

### 2.2 Plasma isolation

Blood samples were collected in plasma collection tubes (EDTA BD Vacutainer 6 mL). Within 1.5 h of collection, isolation of platelet‐poor plasma was performed. First, the EDTA tube was processed at 900 × *g* for 7 min at room temperature, then supernatant was spun at 2500 × *g* for 10 min at room temperature and in some cases an additional spin of 500 × *g* for 10 min was performed. Aliquots of 1.0 mL were stored at −80°C until further processing.

### 2.3 Manual SEC

Manual SEC was used to isolate plasma vesicles for some of the technical experiments and data were used from the earlier cohort as described previously (Drees et al., 2021). In case of the EV‐fractions, SEC‐fractions 9 and 10 (out of 26) were processed and analysed individually in duplicate.

### 2.4 RNA isolation with TRIzol

Total RNA was isolated from SEC fractions using TRIzol (Thermo Fisher Scientific) according to the manufacturer with some modifications. In brief, 0.75 mL TRIzol was added to 0.25 mL SEC fractions, incubated at room temperature for 15 min and then stored at −80°C for at least 3 h. Prior to isopropyl precipitation, 2.5 µg glycogen (Roche) was added. The final RNA pellet was dissolved in 10 µL nuclease‐free water.

### 2.5 Quality‐controlled EV‐miRNA assay protocol

**FIGURE 1:**
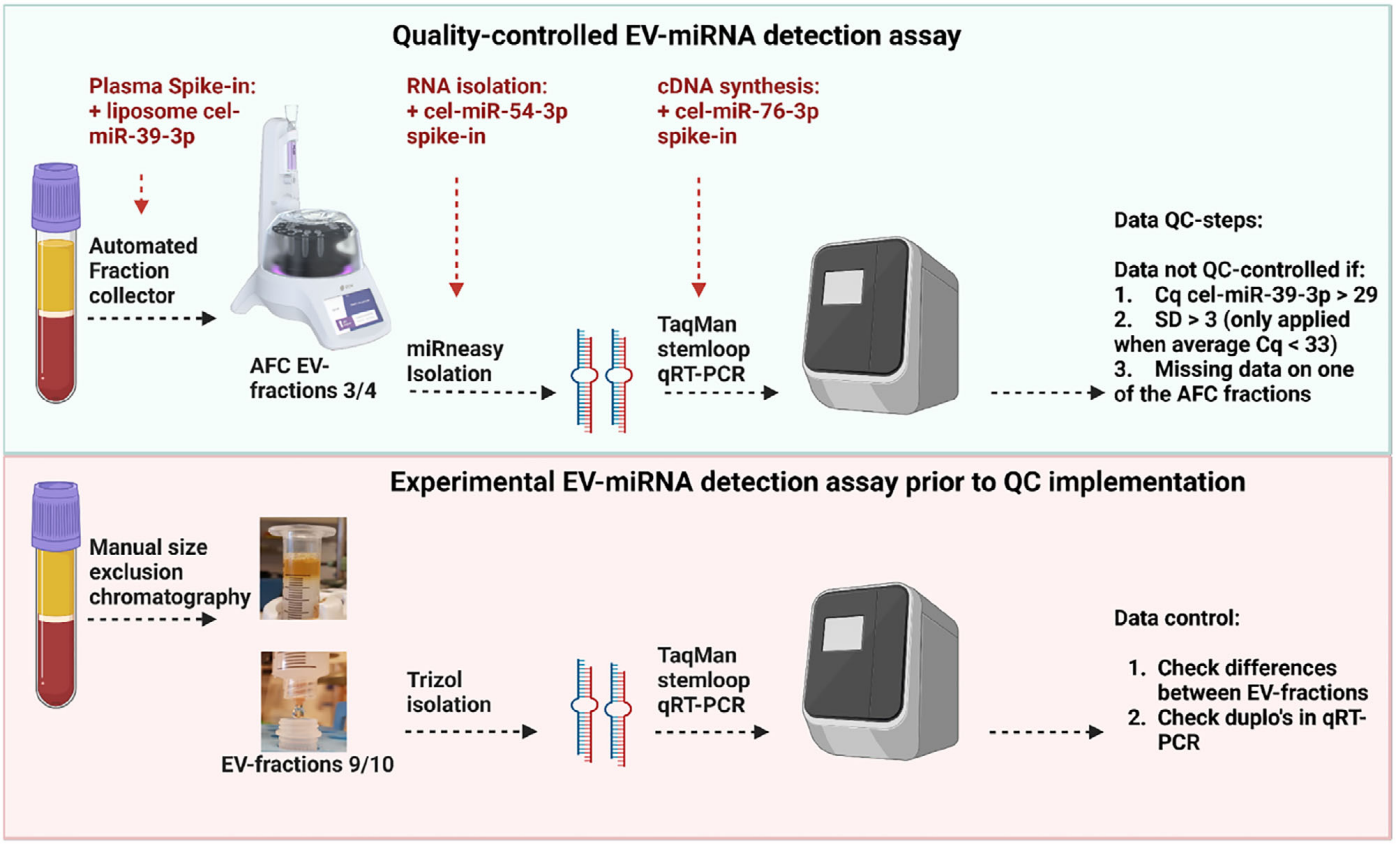
Study schematic. Comparison between the quality‐controlled EV‐miRNA detection assay and the experimental EV‐miRNA detection assay. Platelet‐poor plasma (PPP) from patients was processed from EDTA blood collection tubes. In the quality‐controlled assay (top schematic), liposome‐encapsulated cel‐miR‐39‐3p is added to the plasma, directly followed by bulk EV isolation with size exclusion chromatography (SEC) using an automated fraction collector (AFC). Synthetic cel‐miR‐54‐3p is added to the EV‐enriched fractions and miRNeasy kit was used to isolate the EV‐RNA. Synthetic cel‐miR‐76‐3p was added to purified EV‐RNA to control for cDNA conversion. After multiplex qRT‐PCR, samples that did not adhere to QMS criteria were discarded from further analysis. In the experimental EV‐miRNA detection assay (bottom schematic), manual SEC was used to collect EV‐enriched fractions and TRIzol to isolate EV‐RNA followed by qRT‐PCR. Figure created with BioRender.com. EDTA, ethylenediamine tetraacetic acid; EV, extracellular vesicle; SD, standard deviation; QC, quality control; QMS, quality management system; qRT‐PCR, quantitative real‐time reverse‐transcription PCR.

From 51 cHL patients, 120 plasma samples were analysed in a quality‐controlled EV‐miRNA cHL assay (Figure 1). SEC with the Automated Fraction Collector (AFC‐V1, Izon) was used to isolate plasma vesicles. Prior to isolation, 0.9 mL of plasma was spiked with 50 µL liposome containing *Caenorhabditis elegans* miRNA cel‐miR‐39‐3p (Excytex, cel‐miR39 liposomes, concentration: 1E9 liposomes/µL, Eurogentec, oligonucleotide cel‐miR‐39‐3p: UCA‐CCG‐GGU‐GUA‐AAU‐CAG‐CUU‐G) and 550 µL of phosphate buffered saline (PBS). As input material, 0.9 mL plasma was used as the samples were frozen in 1.0 mL aliquots. The total loaded volume on the column was 1.5 mL. On a qEV original 70 nm column (SP1‐EUR, IZON), using a buffer volume of 2.85 mL, the particles of interest were collected in fractions 3 and 4 both of 0.5 mL (between 1 and 2 mL of the Purified Collection Volume [PCV]). The standard buffer, that is, void volume was advised to us by IZON when setting up protocol. As such, we set up a protocol with 2.85 mL as buffer volume, which is close to the current buffer volume advised by IZON (2.9 mL). Both fractions were analysed individually to serve as an internal duplicate. Post isolation, 200 µL of each fraction was stored in 1000 µL Qiazol (ref. 79306, Qiagen) and frozen at −80°C overnight. RNA from pEVs was isolated using the miRNeasy serum/plasma kit (QIAgen, Cat. No.: 217184) according to the manufacturers' protocol with the addition of glycogen (2.5 µg was added to the upper aqueous phase after the phase separation). Thirty microlitre of the RNA oligo spike‐in cel‐miR‐54‐3p with a concentration of 6.02E5 copies/µL (UAC‐CCG‐UAA‐UCU‐UCA‐UAA‐UCC‐GAG, Eurogentec) was added prior to RNA isolation after thawing of the plasma EVs on ice. Six hundred microlitre of the aqueous phase was used in the precipitation step. MiRNA qRT‐PCR was done as described before (Eijndhoven et al., 2016). As an internal control of the cDNA synthesis, 3 µL of the RNA oligo spike‐in cel‐miR‐76‐3p with a concentration of 6.02E5 copies/µL (UUC‐GUU‐GUU‐GAU‐GAA‐GCC‐UUG‐A, Eurogentec) was added during the cDNA synthesis step. In brief, equal volumes of RNA (3 µL) were reverse transcribed with TaqMan MicroRNA Reverse Transcription kit (Thermo Fisher Scientific). Six RT‐primers (miR‐127‐3p [ID000452], miR‐155‐5p [ID002623], let‐7a‐5p [ID000377], cel‐miR‐39‐3p [ID000200], cel‐miR‐54‐3p [ID001361], cel‐miR‐76‐3p [ID000229]) were used in a validated multiplex reaction. For qPCR, TaqMan Universal PCR Master Mix, No AmpErase UNG (4364343, Thermo Fisher Scientific) was used. This kit contains AmpliTaq Gold DNA Polymerase (Ultra Pure), dNTPs with dUTP, ROX Passive Reference and optimized buffer components. Three microlitre of cDNA was subjected to 40 cycles of 95°C for 15 s and 60°C for 1 min on a Applied Biosystems 7500 Fast system. Data was analysed using 7500 Software v2.3. An auto baseline was used for data analysis. For comparison of miRNA levels, similar Cq threshold levels (0.1) were used in different PCR runs. A QC quality control on the data was performed. Thirteen samples were excluded from further analyses based on the QC data control. Samples did not pass the QC quality control if they met one of the following criteria: (1) Cq cel‐miR‐39‐3p > 29, (2) Duplo's: standard deviation (SD) > 3 of the Cq duplicates (only applied when average Ct < 33) or (3) Missing data on one of the AFC fractions. The setting of the thresholds for the quality‐controlled data was based on the 120 samples analysed with this protocol.

### 2.6 Comparative analyses of the quality‐controlled data and experimental data

For the comparative analysis of the experimental phase protocol and the quality‐controlled EV‐miRNA cHL data, the same data analysis protocol was used. We normalized the qRT‐PCR data to correct for variability in plasma volume input as described in the methods of this paper (Drees et al., 2021). In short, the qRT‐PCR data were normalized to the mean of the complete metabolic response (CMR) samples of the same sample volume so that direct comparison of the different dataset is possible, even though the volume input of the two assay's is different. The relative change, that is, log2‐fold change was calculated using 2–∆∆Cq in which the ∆∆Cq is the difference between the Cq‐value measured at that timepoint minus the mean Cq‐value of the CMR group with the same plasma input. This is to calculate the relative expression of the C *q*‐value in comparison to CMR group, as ∆∆Cq is regularly performed in Cq‐analyses (Bustin et al., 2009; Livak & Schmittgen, 2001; Mestdagh et al., 2009).

Generalized Linear Mixed‐Effects model (GLMM, R‐package lme4 [Bates et al., 2015]) with bootstrapping is used to test clinical performance of the QC‐EV‐miRNA assay. The data of the quality‐controlled EV‐miRNA assay was subsequently compared to the experimental EV‐miRNA assay's data (Drees et al., 2021), which was re‐analysed using the same GLMM method with bootstrapping. A patient random effect was included in the model to account for the repeated measurements within patients. The same three EV‐miRNAs were used in both assay's as biomarkers. Bootstrapping was used to calculate the optimism‐corrected performance (in terms of the AUC) (Efron & Tibshirani, 1993; Harrel, 2015; Neeman, 2009; Van Houwelingen & Le Cessie, 1990). Youden‐index was used to determine the sensitivity, specificity, negative predictive value and positive predictive value (Bates et al., 2015). The pROC R package was used to produce the receiver operating characteristic (ROC)‐curves and calculate the AUC (Robin et al., 2011). Prior to the GLMM, we performed a so‐called test‐retest analysis with a logistic regression to validate the QC steps (data not shown). However, because this type of analyses does not correct for repeated measurements within one patient, the GLMM‐analyses was used to compare the clinical performance between the experimental‐ and QC data.

### 2.7 Study approval

Samples were collected in the BioLymph‐study (2017‐2019, VUmc METc registration number: 2017.008). The study was registered in the Dutch ‘centrale commissie mensgebonden onderzoek’ CCMO‐register (toetsingonline.nl, NL60245.029.17) and is being conducted in accordance with the Declaration of Helsinki (7th revision, October 2013) and the Medical Research Involving Human Subjects Act (WMO). A second set of samples (2014–2017), prior to the BioLymph study, was collected in a biobanking protocol approved by the Amsterdam VUmc Biobank Approval Committee (registration number 2018.359) and approval for use in the present study granted (registration number U2018.044).

## 3 RESULTS

Design of a spike‐in controlled EV‐miRNA assay to determine efficiencies of EV recovery from stored plasma, EV‐RNA isolation, cDNA synthesis and amplification of miRNAs

In order to design a practical quality‐controlled assay for EV‐miRNA detection, we amended our experimental assay (Drees et al., 2021) of which the differences are depicted in Figure 1. In the quality‐controlled assay, EV‐isolation was performed with the qEV, a commercial SEC column (IZON) and with an AFC device (Izon) instead of manually prepared columns. Liposomes loaded with C. elegans encoded miRNA cel‐miR‐39‐3p were added to patient plasma prior to isolation with AFC as to control this step of the assay. With quality‐control, RNA was isolated from AFC fractions 3 and 4 with the miRNeasy method instead of the TRIzol RNA isolation method we used in the experimental assay. C‐elegans miRNA cel‐miR‐54‐3p was added to the plasma prior isolation to control for RNA isolation efficiency. To detect technical variance in the final qRT‐PCR step, C. elegans miRNA cel‐miR‐76‐3p was added during the cDNA‐synthesis. All three C. elegans spike‐ins were included in the multiplex qRT‐PCR detection with our three endogenous miRNAs (miR‐155‐5p, miR127‐3p and let‐7a‐5p). Prior to approving the data for interpretation, samples were excluded with too much technical variation between duplicates.

**FIGURE 2:**
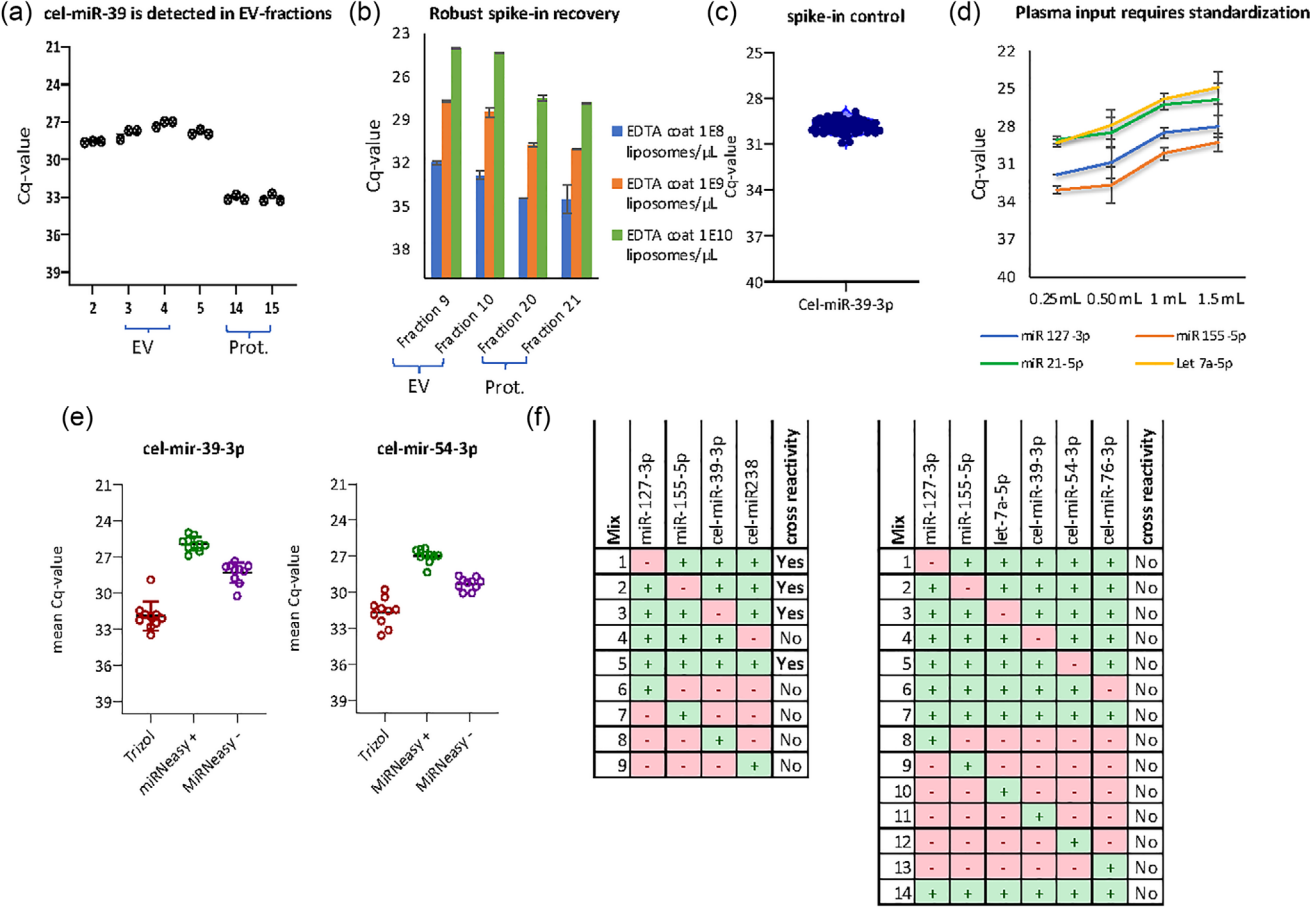
Design of a quality‐controlled EV‐miRNA assay with synthetic spike‐ins to quantify technical noise. (a) Liposomes containing cel‐miR‐39‐3p were added to plasma and EVs were isolated with an AFC device. Cel‐miR‐39‐3p levels were measured by qRT‐PCR in the EV‐fractions 2–5 and protein‐fractions 14, 15. The mean of the duplo's are shown with standard deviations. (b) Liposomes encapsulated with cel‐mir‐39‐3p were added at three different concentrations into PBS containing EDTA. Cel‐miR‐39‐3p levels (measured in duplicate) are shown in EV‐enriched fractions 9 and 10 and protein fractions 20 and 21. The mean of the duplo's are shown with standard deviations. (c) Cel‐miR‐39‐3p was measured with qRT‐PCR in 86 cHL plasma samples using the experimental EV‐miRNA protocol with standard 1.0 mL plasma input. Of note, the measurements were done by three different operators over a timespan more than 1 year. (d) Plasma from a healthy donor was divided in four different volumes. EVs were enriched with SEC and endogenous miR‐127‐3p, miR‐155‐5p, miR‐21‐5p and let‐7a‐5p were quantified with qRT‐PCR. Fractions were measured in duplo and the mean of the two EV‐fractions are shown in the plot. (e) AFC EV‐fractions 3–4 were split into 10 equal volume (1.0 mL) samples and RNA was isolated with either TRIzol or miRNeasy with and without glycogen (i.e., +/‐). The bars represent the mean Cq value from qRT‐PCR measurements (in duplo) of cel‐miR‐39‐3p and cel‐miR‐54‐3p. (f) Leave‐one‐out cross validation is used to determine the efficiency of individual qRT‐PCRs in a combined (multiplex) cDNA reaction. Shown are the different primer mixes tested in a table. + means the primer was present in the mix and cross reactivity is shown in the last column. The full mix contains miR‐127‐3p, miR‐155‐5p, let‐7a‐5p and two spike‐in controls: cel‐miR‐39‐3p, cel‐miR‐238. As input RNA from KMH2 (Hodgkin) cell line is used. AFC, automated fraction collector; EDTA, ethylenediamine tetraacetic acid; EV, extracellular vesicle; PBS, phosphate buffered saline; qRT‐PCR, quantitative real‐time reverse‐transcription PCR.

To validate the spike‐ins as assay controls we performed several experiments. Pooled plasma from multiple donors was run in triplicate on the AFC and liposome spike‐in qRT‐PCRs were performed for multiple fractions (Figure 2a). AFC fractions 3 and 4 clearly contained the highest levels of synthetic cel‐mir‐39‐3p spike‐in and endogenous miRNA let‐7a‐5p, typically highly enriched in plasma EVs (Eijndhoven et al., 2016), corresponding with the EV‐enriched fractions of the AFC (Figure 2a). Serial dilution experiments of the liposome spike‐in were performed to determine an optimal concentration corresponding to a Cq value of approximately 30 (Figure 2b). At this level duplo's are reliable while small variations in pipetting are not exaggerated. Robust recovery of cel‐miR‐39‐3p was detected in 86 cHL samples measured with the experimental assay when using 1.0 mL input by different operators at different timepoints over a timespan of more than a year (Figure 2c). These results support the robustness of EV‐RNA recovery confirming the liposome spike‐in cel‐miR‐39‐3p as suitable control for the EV‐isolation step. Furthermore, we observed that the level of EV‐miRNAs measured by qRT‐PCR is determined by the plasma input volume (Figure 2d). Thus supporting the validity of a quality‐ controlled quantitative assay based on plasma input volume for normalization between samples. The experimental assay data was obtained with 1.0 or 1.5 mL plasma as input volume. For practical reasons, in the quality‐controlled EV‐miRNA protocol we standardized the plasma‐input volume to 0.9 mL. In our experience, plasma volume in frozen aliquots did not always equal 1 mL.

To validate the methodology for miRNA isolation from AFC‐generated EV fractions we compared the efficiency of the TRIzol and miRNeasy protocols, with and without addition of glycogen (often applied to increase RNA recovery). RNA from pooled plasma EV from three different donors was isolated 10‐times with miRNeasy with and without glycogen and compared to the original RNA isolation using TRIzol reagent including glycogen. The miRNeasy protocol yielded a higher recovery of EV‐miRNAs (Figure 2e). The addition of glycogen increased the RNA yield and improved the detection of all endogenous miRNAs (Figure 2e) as well as the RNA‐spike‐in control cel‐miR‐54‐3p (Figure 2e). We designed multiplex cDNA conversion reactions of the EV‐RNA using leave‐one out cross‐validation. This led us to exclude cel‐miR‐238 as spike in as it yielded irregular results suggesting cross‐reactivity of the primers (Figure 2f). Additionally, cel‐miR‐238 levels had a lower rate of recovery in the samples (high Cq), making it less attractive as a spike‐in. As such, cel‐miR‐76‐3p was selected as spike‐in control for the cDNA synthesis step. The full leave‐one out cross‐validation of the final multiplex primer set is depicted in Figure 2f. After these adjustments we called a ‘design freeze’ of the optimal EV‐miRNA assay.

### 3.1 Quality‐controlled assay and clinical performance

**FIGURE 3:**
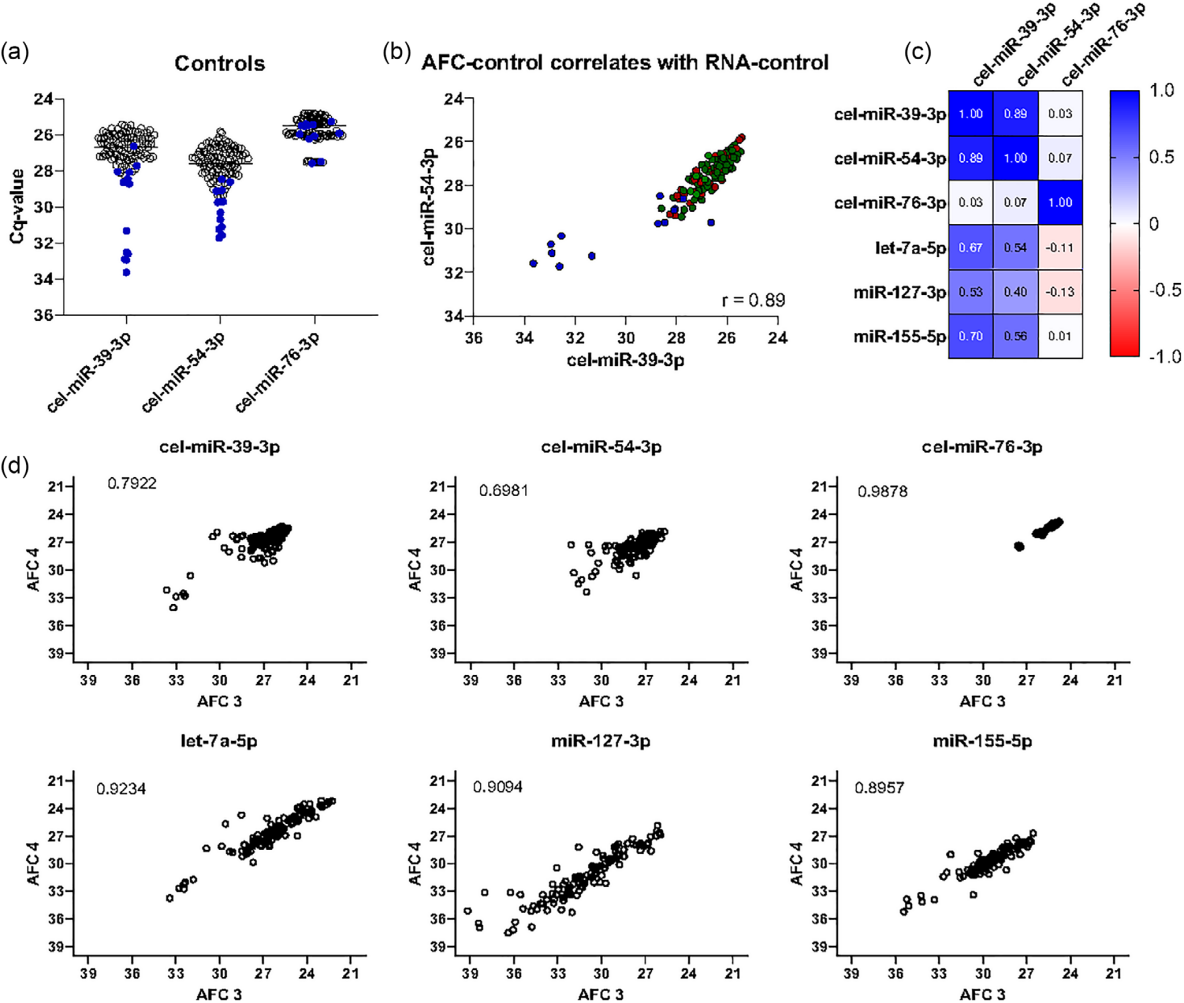
Internal quality controls increase the reliability of the data. (a) qRT‐PCR of the spike‐in controls as detected in the 120 QMS cHL samples. Samples in blue failed to pass QMS quality control criteria (see top right Figure 1). (b) Correlation between cel‐mir‐39‐3p and cel‐mir‐54‐3p (Cq‐values). Samples in blue did not pass quality control. In green the samples from patients with a complete metabolic response and in red samples from patients with FDG‐PET confirmed active disease. (c) A heatmap of the calculated correlation coefficient r (Pearson) between spike‐in and endogenous miRNAs. (d) Correlation between AFC EV‐fractions 3 and 4 AFC for all three spike‐ins and miR‐127‐3p, miR‐155‐5p and let‐7a‐5p. Correlation coefficient *r* (Pearson) is depicted in the graph. All 120 samples are included, including the samples that did not pass quality control. AFC, automated fraction collector; cHL, classic Hodgkin lymphoma; EV, extracellular vesicle.

Having optimized the design of the quality‐controlled assay for technical performance, 120 plasma samples from 51 cHL patients were analysed using this protocol (Figure 1). Thirteen samples did not pass quality control based on strongly aberrant (cut‐off > Cq > 29) cel‐miR‐39‐3p values (*n *= 12) or when data was missing in one of the AFC fractions (*n* = 1). The threshold for cel‐miR‐39‐3p was set on the data shown in Figure 3a. However, no samples met the exclusion criterion of a high standard deviation between the Cq values of the different PCR duplicates (SD of > 3). In total 90% of the samples passed QC without repeating samples or analyses.

Figure 3a depicts the exogenous controls of the 120 samples, indicated in blue are those samples that did not pass quality control. We observe a strong correlation between cel‐miR‐39‐3p and cel‐miR‐54‐3p (*r* = 0.89, Figure 3b) levels but not with cDNA spike‐in (cel‐miR‐76‐3p), indicating that most technical variability is introduced during the RNA isolation step (Figure 3c). As such, there was no need for an absolute threshold for both spike‐ins (cel‐miR‐39‐3p and cel‐miR‐54‐3p) as this does not alter the number of samples included in the analyses. There is a strong correlation (0.70 to 0.99) in miRNA levels between AFC fractions 3 and 4 for both the endogenous and spike‐in miRNAs, indicating robustness in EV isolation recovery by SEC when using the AFC device (Figure 3d).

### 3.2 Direct comparison between quality‐controlled EV‐miRNA measurements and experimental phase EV‐miRNA assay

**FIGURE 4:**
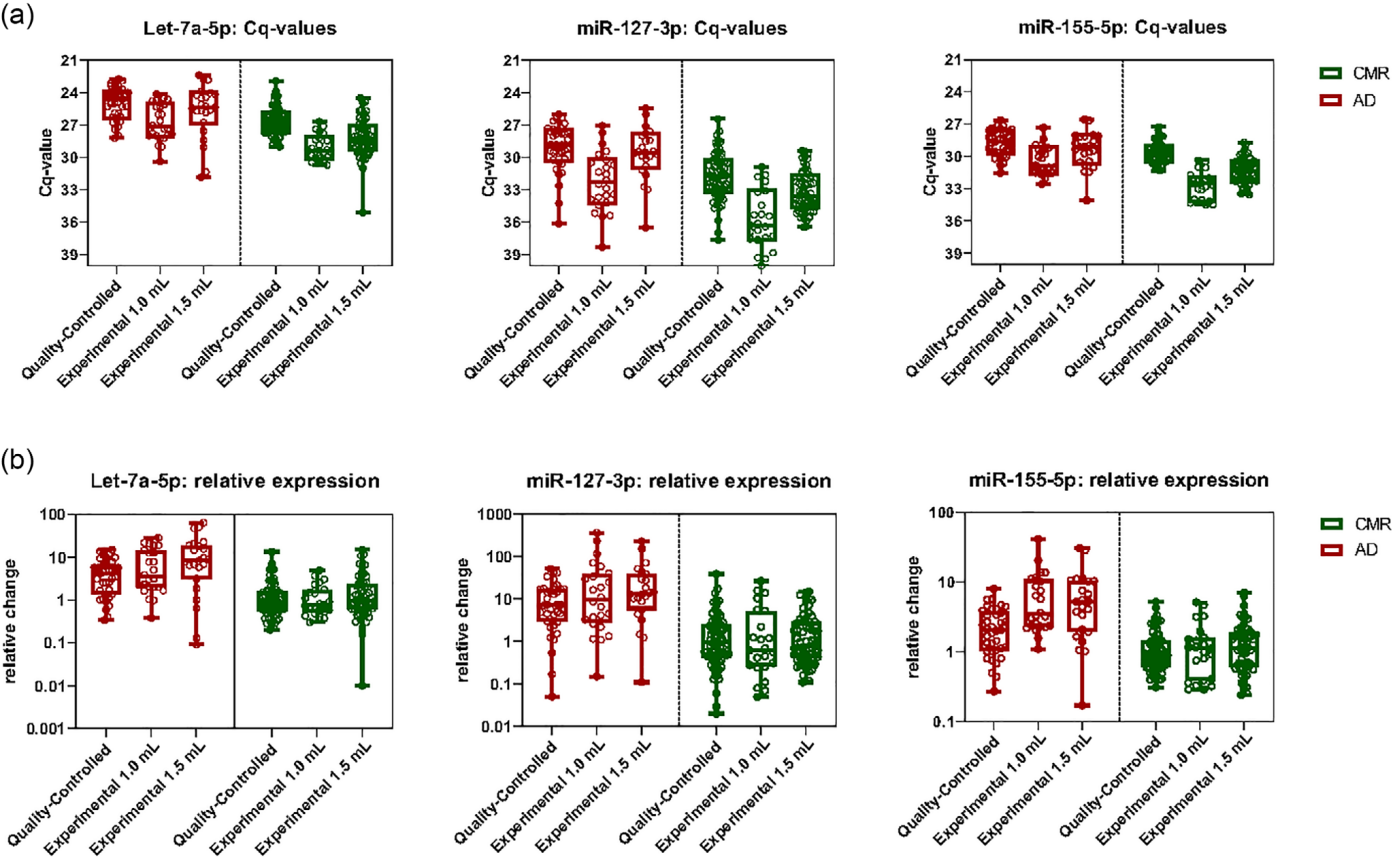
Normalization allows for data comparison between different plasma‐inputs. (a) Raw Cq‐values of the qRT‐PCR analysis of EV‐associated miR‐127‐3p, miR‐155‐5p and let‐7a‐5p in samples from patients with active (FDG‐PET positive) disease (AD in red) with versus those with complete metabolic response (CMR in green) split over the different type of analyses and sample input. (b) as in (a) but represented as relative expression (log2‐fold change) levels. Whiskers represent min and maximum values. Each point is one patient sample. AD, active disease; CMR, complete metabolic response; EV, extracellular vesicle.

To understand the effect of the adjustments on the diagnostic performance we compared the quality‐controlled data to the experimental EV‐miRNA assay (Figure 4). As expected, Cq‐values for all miRNAs were on average lower with 1.0 mL input compared to 1.5 mL. In addition, the change in RNA‐isolation method (TRIzol vs. miRNeasy) applied in the quality‐controlled EV‐miRNA protocol increased miRNA detection level. As a consequence, the absolute abundance of miRNAs in the quality‐controlled data was higher than that obtained with the experimental data (Figure 4a), precluding direct comparison of the assay outcome without normalization.

**FIGURE 5:**
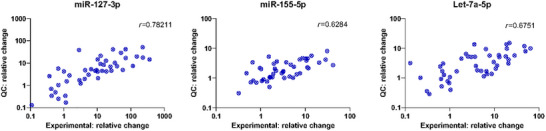
Direct comparison between quality‐controlled EV‐miRNA measurements and experimental EV‐miRNA assay. Correlation between experimental assay qRT‐PCR measurements and the quality‐controlled data. The *y*‐ and *x*‐axis represent relative expression (log2‐fold change). A spearman correlation statistic was used to calculate the correlation coefficient (*r*). Each dot is one sample. EV, extracellular vesicle; QC, quality control; qRT‐PCR, quantitative real‐time reverse‐transcription PCR.

**FIGURE 6:**
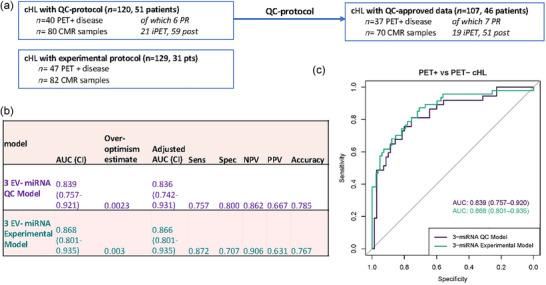
Quality‐controlled EV‐miRNA assay reproduces performance of the experimental assay. (a) Set up of the comparative analyses of the quality‐controlled (QC) EV‐miRNA assay and the experimental EV‐miRNA assay (Drees et al., 2021). Generalized linear mixed effects models (GLMM) with bootstrapping (to correct for overestimation) is used to calculate and compare the diagnostic performance. (b) Table with the area under the curve (AUC), sensitivity (Sens), specificity (Spec), accuracy, negative predictive value (NPV), positive predictive value (PPV), overoptimism estimate and adjusted AUC of the GLMM‐models with bootstrapping. Cut‐off to determine sensitivity and specificity was performed making use of the Youden‐index. (c) ROC curve generated by GLMM of both the QMS EV‐miRNA assay and experimental EV‐miRNA assay. cHL, classic Hodgkin lymphoma; CI, confidence interval; EV, extracellular vesicle.

By normalizing the datasets (Figure 4b), we could directly compare the quantitative results of the experimental assay with the results from the quality‐controlled EV‐miRNA assay (*n* = 45). We observed positive correlations for all three endogenous EV‐miRNAs, miR‐127‐3p (*r* = 0.782), miR‐155‐5p (*r* = 0.629) and let‐7a‐5p (*r* = 0.675, Figure 5). Inevitable technical differences between the assays, operators may explain deviances between the qRT‐PCR values per sample. To study whether the normalization procedure negatively affects the clinical outcome (classification), we analysed the quality‐controlled EV‐miRNA cHL cohort both with and without calculating relative expression prior to GLMM modelling with bootstrapping (Figure S1a). Despite a strong correlation between the raw Cq and relative expression data (*r *= 0.875); sample classification (active disease [AD] vs. CMR) changed in 10% (11 out of 107) of the samples that passed QC. The overall clinical performance was however virtually unaffected, as both models with either relative expression and Cq‐values yielded similar areas under the curve (AUCs) (AUC = 0.839 versus 0.836, Figure S1b,c and Figure 6). Thus, on a group level the normalization did not affect the clinical performance of the assay but classification changed for some individual samples. We concluded that for quantitative EV‐miRNA assays it is recommended to standardize plasma volume input.

### 3.3 Quality‐controlled EV‐miRNA assay reproduces performance

The QC‐approved cHL EV‐miRNA data (*n* = 107) and experimental assay cHL dataset (*n* = 129) were analysed with GLMM with bootstrapping to calculate the overoptimism estimate and confidence intervals (Figure 6a). A three EV‐miRNA cHL model, comprised of let‐7a‐5p, miR‐127‐3p and miR‐155‐5p, achieves an AUC of 0.87 (confidence interval [CI]: 0.80–0.94) with a minimal overoptimism estimate (Figure 6b,c), a high sensitivity (87.2 %) and intermediate specificity (70.7%). The QC‐approved data (Figure 6b,c) has a similar clinical performance with an AUC of 0.84 (CI: 0.76–0.92), sensitivity of 75.7% and specificity of 80.0%. The accuracy was slightly higher (78.5% vs. 76.7%). These results show that we could validate the clinical performance of quality‐controlled EV‐miRNAs assay, paving the way for future technical and clinical validation.

## 4 DISCUSSION

Circulating miRNAs as biomarkers were proposed over a decade ago (Arroyo et al., 2011) but due to biological (low sensitivity of single markers) and technical hurdles (isolation, detection, noise) their clinical utility has been limited. We developed and validated a quality‐controlled EV‐miRNA qRT‐PCR assay. We compared the diagnostic performance of the quality‐controlled assay with that of our previously generated experimental data (Drees et al., 2021) and show that EV‐bound miRNA quantification is a robust and reproducible liquid biopsy strategy. Our quality‐controlled EV‐miRNA data yields an equally solid diagnostic performance in detection of FDG‐PET positive disease in cHL patients.

Quantifying and controlling the technical noise in the measurements allows for evaluating the true clinical potential of diagnostic assays. In a previous study we showed that measurements of miRNAs bound to plasma EVs can differentiate cHL patients from healthy controls while this was not the case when measuring miRNAs in total plasma (Eijndhoven et al., 2016). Our study set‐up was inspired by Valihrach et al. (2020) making use of exogenous synthetic miRNA spike‐ins to dissect biology‐motivated variation in plasma EV‐miRNA levels from technical noise. The use of endogenous controls alone is inadequate due the vast heterogeneity of plasma EVs and intra‐ or interpatient variability. In our assay, variation in liposome‐associated cel‐miR‐39‐3p and cel‐miR‐54‐3p was observed but almost no variation in cel‐miR‐76‐3p (Figure 2a). This illustrates that the technical noise introduced in the assay is mostly the result of variable EV RNA‐isolation efficiency, rather than EV isolation or cDNA conversion.

Possibly, the diagnostic performance of the quality‐controlled EV‐miRNA assay can be further improved. First, the technical noise introduced during the EV‐RNA isolation step might be avoided when using a different method. For example one may opt for an automated isolation technique such as Kingfisher (ThermoFisher) or Maxwell (Promega) systems. Second, the input volume for the quality‐controlled EV‐miRNA assay was set at 0.9 mL due to availability restrictions. We observed that the difference in Cq‐values between AD and CMR is more robust when input levels are higher, that is, 1.5 mL (Figure 4). The input level is particularly relevant for robust detection of lowly abundant lymphoma‐associated miRNAs such as miR‐127‐3p (Figure 4). Third, residual platelets that carry these miRNAs may cause additional unwanted variation and may be removed with a simple filter in the SEC column as we recently showed (Bracht et al., 2023). Lastly, normalization for input was needed to directly compare the quality‐controlled data with the experimental data. Although the diagnostic performance at a group level (i.e., AUC) did not change, in 10% of the cases, the samples (Figure S1a) were classified differently. To make sure that classification is based on similar performance of the assay, we recommend to maintain a strictly standardized input volume of 1.5 mL (if available) for quantitative EV‐miRNA assays, making the need for standardization against a CMR‐group of samples obsolete.

What are the potential implications of this study for cHL clinical care? cHL is a relatively rare lymphoid malignancy with a good prognosis when treated with appropriate regimens of polychemotherapy (Driessen et al., 2021). Improving treatment outcome relies on personalized approaches with de‐escalation of standard of care treatment for low risk patients to avoid unnecessary (late) treatment‐induced toxicities. Alternative intensification or alternative treatment approaches may be considered for (very) high risk patients. Pre‐ and during‐treatment biomarker monitoring is key to support clinical decision‐making. With an AUC of 0.84 (CI: 0.76–0.92), a sensitivity of 75.7%, a specificity of 80.0% and an accuracy of 78.5% (Figure 6) for predicting disease response in cHL, the EV‐miRNA assay alone is unlikely to replace FDG‐PET monitoring. However, in combination with ELISA‐based detection of the serum chemokine TARC, the negative predictive value (NPV) for detecting complete metabolic response (i.e., FDG‐PET‐negative) may reach the level of clinical utility as shown in our prior experimental study (Drees et al., 2021). Implementation of a combined EV‐miRNA/TARC assay may individualize cHL treatment to reduce (long‐term) side effects in low risk patients. The clinical performance of this cHL EV‐miRNA assay should be evaluated further for technical and clinical validation to become fully IVDR‐compliant.

## AUTHOR CONTRIBUTIONS

**Esther E. E. Drees**: Conceptualization; data curation; formal analysis; investigation; methodology; resources; software; visualization; writing—original draft. **Nils J. Groenewegen**: Data curation; formal analysis; investigation; methodology; validation. **Sandra A. W. M. Verkuijlen**: Resources. **Monique A. J. van Eijndhoven**: Resources. **Jip Ramaker**: Investigation. **Pepijn Veenstra**: Investigation. **Mirjam Hussain**: Investigation. **Catharina G. M. Groothuis‐Oudshoorn**: Formal analysis; methodology. **Daphne de Jong**: Investigation; writing—review and editing. **Josée M. Zijlstra**: Data curation; investigation; resources; writing—review and editing. **Johan de Rooij**: Conceptualization; Data curation; formal analysis; funding acquisition; investigation; methodology; project administration; supervision; writing—review and editing. **Dirk M. Pegtel**: Conceptualization; data curation; formal analysis; funding acquisition; investigation; methodology; project administration; supervision; validation; visualization; writing—original draft; writing—review and editing.

## CONFLICT OF INTEREST STATEMENT

D.M.P. is co‐founder, shareholder and CSO of Exbiome BV, receives funding from the Stichting MRD in Hodgkin Lymphoma, has served as an advisor for Takeda and received research funding (Intl. Scholars Award in hemato‐oncology) from Gilead and is a consultant for Y2Y. D.M.P. is an inventor on a patent related to EV‐RNA diagnostics submitted by Johns Hopkins University. The other authors declare no conflicts of interest. ExBiome measured the plasma samples and developed the QMS‐compliant assay but was not part of the analyses that determined the clinical performance of the data and was not given access to clinical data and context of the samples.

## Supporting information

Supporting Information

Supporting Information

## Data Availability

Proposals for data sharing should be directed to d.pegtel@amsterdamumc.nl; to gain access, data requestors will need to provide a draft of a data access agreement that will be evaluated.
