# Supplementary material for: Towards IVDR‐compliance by implementing quality control steps in a quantitative extracellular vesicle‐miRNA liquid biopsy assay for response monitoring in patients with classic Hodgkin lymphoma
Source: J Extracell Biol. 2024 Jun 28;3(7):e164. doi: 10.1002/jex2.164 (PMC11213689; doi:10.1002/jex2.164)
Supplement: Supplementary file 1 — Supporting Information [file JEX2-3-e164-s002.pdf]

Suppl. Figure 1: Quality-controlled EV-miRNA assay analyzed with GLMM with bootstrapping with Cq-values

A

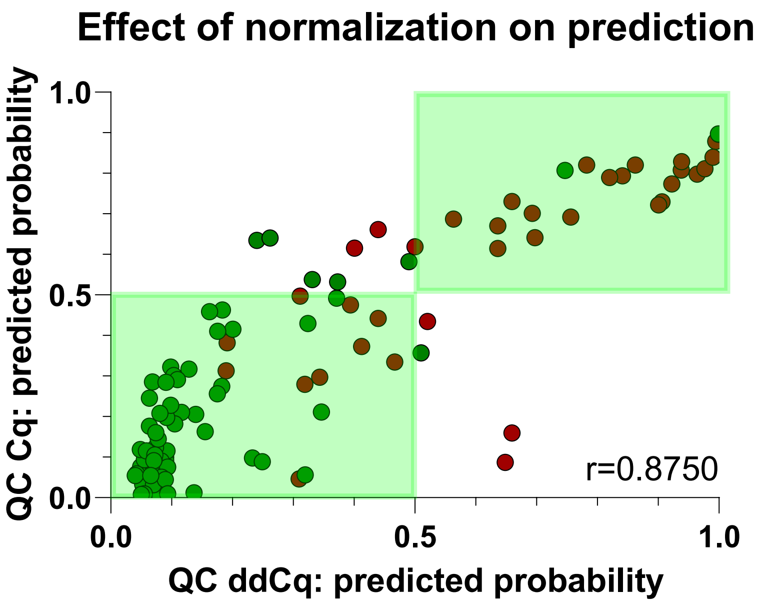

C

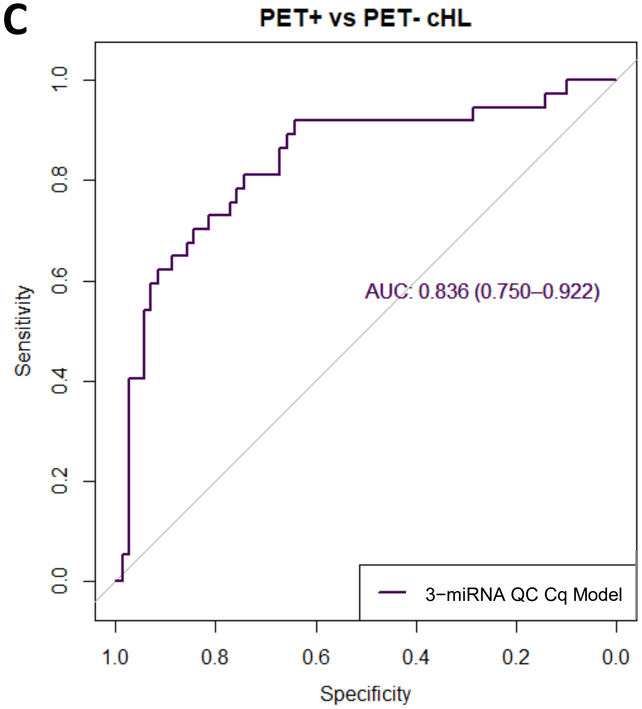

B

	AUC (CI)	Over-optimism estimate	Adjusted AUC (CI)	Sens	Spec	NPV	PPV	Accuracy
3 EV- miRNA QC Cq model	0.836 (0.750-0.922)	0.000	0.836 (0.750-0.922)	0.919	0.642	0.938	0.576	0.738
