# Supplementary material for: Towards IVDR‐compliance by implementing quality control steps in a quantitative extracellular vesicle‐miRNA liquid biopsy assay for response monitoring in patients with classic Hodgkin lymphoma
Source: J Extracell Biol. 2024 Jun 28;3(7):e164. doi: 10.1002/jex2.164 (PMC11213689; doi:10.1002/jex2.164)
Supplement: Supplementary file 2 — Supporting Information [file JEX2-3-e164-s001.pptx]

## Slide 1
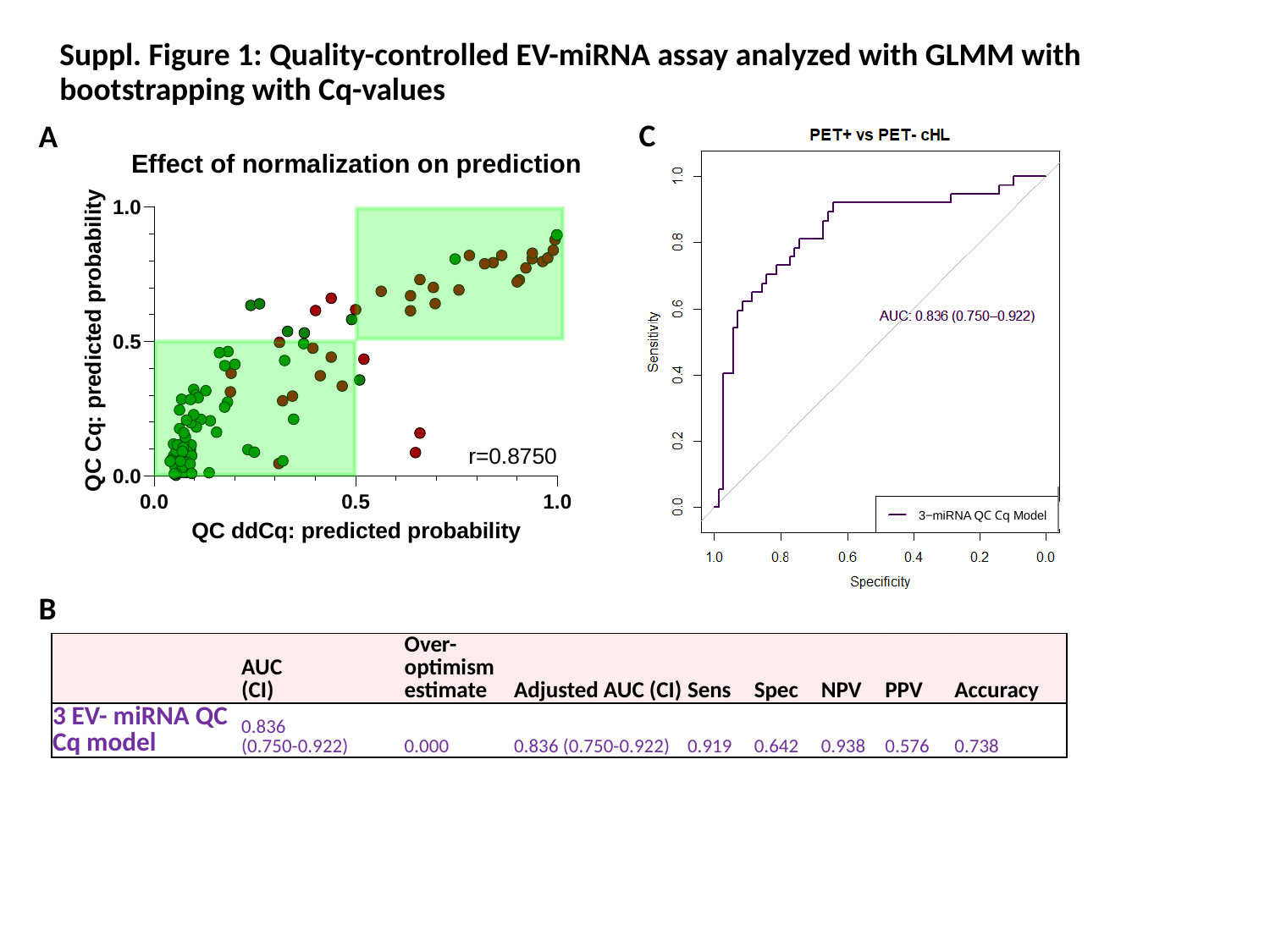

Suppl. Figure 1: Quality-controlled EV-miRNA assay analyzed with GLMM with bootstrapping with Cq-values
C
A
3−miRNA QC Cq Model
B
| | AUC (CI) | Over- optimism estimate | Adjusted AUC (CI) | Sens | Spec | NPV | PPV | Accuracy |
| --- | --- | --- | --- | --- | --- | --- | --- | --- |
| 3 EV- miRNA QC Cq model | 0.836 (0.750-0.922) | 0.000 | 0.836 (0.750-0.922) | 0.919 | 0.642 | 0.938 | 0.576 | 0.738 |
